# Determinants of traditional medicine utilization for children: a parental level study in Tole District, Oromia, Ethiopia

**DOI:** 10.1186/s12906-020-02928-1

**Published:** 2020-04-23

**Authors:** Fekensa Hailu, Amsale Cherie, Tigistu Gebreyohannis, Reta Hailu

**Affiliations:** 1 School of Medicine, Jimma University, Jimma, Ethiopia; 2 College of Health Sciences, School of Nursing and Midwifery, Addis Ababa University, Addis Ababa, Ethiopia; 3 Faculty of Environment, Gender, and Development Studies, Hawassa University, Hawassa, Ethiopia

**Keywords:** Traditional medicine, Children, Parents, Tole District, Ethiopia

## Abstract

**Background:**

In Ethiopia, about 80% of the population use traditional medicine (TM) due to the cultural acceptability of healers and local pharmacopeias, the relatively low cost of traditional medicine, and the difficulty of accessing modern health facilities. This study was aimed at assessing traditional medicine utilization and its determinants among parents of the children employing a case study of the Tole District of South West of Oromia, Ethiopia.

**Methods:**

A community-based cross-sectional data were collected from 267 parents who have children less than 18 years old. The respondents were selected through a systematic random sampling technique. Both descriptive and exploratory techniques were used to analyze the data. The exploratory logistic regression analysis was carried out to identify factors determining the use of traditional medicine (TM).

**Results:**

We found out that 85.9% of parents used TM for their children. Herbal medicine 73 (34.4%), massage 55 (25.9%), and religious/prayer therapy 25 (11.8%) were the major therapies used by parents for their children. In the study area, the rate of parental TM utuilization for their children was determined by monthly income [OR: 0.25(0.08, 0.78)], cultural belief [OR: 3.01(1.16, 7.83)], religious belief [OR = 3.17(1.26, 7.93)], and duration of illness [OR = 3.11(1.07, 9.02)].

**Conclusion:**

Traditional medicine use is highly prevalent that its contribution to the public health is significant as some could not access to and afford modern health services in the area. Therefore, health professionals should advise parents side-by-side procuring modern health services. In light of this, further research will be needed on the safety and efficacy of TM for wider application.

## Background

The importance of traditional medicine as a source of primary healthcare was first officially recognized by the World Health Organization (WHO) in the Primary Health Care Declaration of Alma Ata in 1978 [1]. Traditional medicine (TM) is defined as “*the total of the knowledge, skills, and practices based on the theories, beliefs, and experiences indigenous to different cultures, whether explicable or not. It is used in the maintenance of health as well as in the prevention, diagnosis, and treatment of physical and mental illness*” [2]. It includes the use of the plant, animal and mineral-based medicines, spiritual therapies, and manual techniques and exercises applied singularly or in combination.

The history of TM is as long as the history of human beings in this world. Historical evidence revealed that apparently, all primitive peoples have used some forms of TM since longtime. Study shows that several countries in Africa, Asia, and Latin America have been using TM to meet some of their primary health care needs [3]. In China, the medicinal herb, *Artemisia annua*, was used for almost 2000 years and found to be effective against resistant malaria [4]. In Nigeria, Quinine from plant cinchona bark was used to manage the symptoms of malaria long before the disease was identified. Moreover, a willow bark, a garden aspirin tablet, has been popular painkiller very long before we have had access to tablet making machinery [5].

In customarily African society, traditional practitioners are responsible for re-establishing social and emotional equilibrium based on traditional community rules and relationships [6]. TM users typically view sickness as the failure of complex social and spiritual relationships. However, the disease burden is growing rapidly that indigenous African medicine can be used as an alternataive and affordable remedies within the reach of millions who are unable to access modern healthcare as it is expensive or inaccessible. In other words, TM provides an alternative medicine for those who prefer, for many reasons, to be treated in a more culturally sympathetic and familiar way [7–9].

Studies estimated that some 80% of Ethiopians are relying on traditional healers and remedies for their healthcare needs for centuries, which is vastly complex, diverse, and varies greatly among different ethnic groups [10]. Even though traditional medicine plays an important role among the Ethiopian society, the knowledge and understanding on the extent and characteristics of traditional medical practices are limited. So far, the national health system neither studied the full therapeutic potential of TM nor estimated that the accurate potential adverse effects. Moreover, the types of TM and factors determining utilization were not thoroughly and scientifically studied at the community level [11]. TM in Ethiopia is not uniformly practiced. The uses are considerably diverse and significantly vary between regions. The standard policies and regulations governing TM are too weak or do not exist at all. Instead, the users associated with cultural beliefs. Consequently, TM is an integral part of a community’s identity and value that it is difficult to regulate through a nationwide framework concerning its safety and effectiveness using different medicine categorizations and definitions [12–14].

As such, the prevalence and utilization, as well as factors determining TM use for all categories of population in general and pediatrics in particular are not adequately studied and documented. To this end, this study aimed at assessing the TM utilization for children and associated factors among parents of children in the Tole District, Southwest Shewa Oromia Regional State, Ethiopia. The study helps to improve child health practice and identify the possibilities that the professional health service providers advise for the community in utilizing TM hand-in-hand with procuring modern health services.

## Methods

### Study participants

The study employed a cross-sectional data. We collected data in March 2017 from Tole District. The District is found in Southwest of Shewa, Oromia Region of Ethiopia about 80 km in the Southwest of Addis Ababa. The District has two urban and 24 rural kebeles (the lowest administrative unit in Ethiopia). The total estimated population is about 82,377 (44,653 males and 40,724 females). It has 15,078 households and 13,535 children under 5 years old. The majority of the people (90.87%) are believers of Orthodox Christians. The District has 4 Health Centers, 24 Health Posts, 7 Private Clinics, and 2 Private Drug Stores [15].

The sample size for this study was calculated using single population proportion formula considering the following assumptions: prevalence of traditional medicine use for children 88.2% [3], with 5% margin of error, 95% Confidence Interval (CI), design effect of 1.5, and 10% of non-response rate. Accordingly, 267 parents who have children < 18 years old were selected using a systematic random sampling technique. The selection process was followed pre-data collection assessment, screening, and numbering. The calculated sample interval was 12. After the random selection of the first household, every 12^th^ of household was selected. In case this was not possible due to any reason, the immediate next parent was considered for the same.

Consequently, data were collected from sampled parents, who had children < 18 years of age, who lived in the Tole District for at least 6 months, parents/guardians who lived with the children, and available at the time of data collection. However, parents who were seriously ill or unable to give the required information during the data collection period were excluded.

### Instruments

A structured questionnaire was used for the data collection. The tool was administered by trained enumerators. During data collection, either the father or mother of the children was interviewed. Nevertheless, the priority was given to mothers because they are closer to their children than fathers. Still, when the mother was not available by any means, the father was interviewed. The questionnaire was adapted from previous research done on similar topics in Ethiopia [3] and was translated into the local language (Afan Oromo). Consistency and equivalency was checked by translating the Afan Oromo version back to English by another individual who was fluent in both languages. The questionnaire constituted six parts. The first part comprised of the socio-demographic characteristics of parents. The second part included the traditional medicine practice and utilization. The third part outlined the type of TM. The fourth and fifth sections addressed the socio-cultural environment and the perception of illness or sickness of the parent, respectively. The final section of the questionnaire was about healthcare experience of the respondents (see supplementary material).

The data was collected by a team composed of 10 Health Extension Workers (HEWs), five-degree level graduate nurses, and two supervisors. Before collecting data, they were trained on the purpose and objective of the survey, the content of the questionnaire, meaning of each question, and the approach to conduct the interview and to record the response. The data collectors were assigned out of their respective duty stations to minimize bias. In advance of the actual interview, a pre-test was done on 14 non-sampled respondents (5% of intended respondents) to check the validity and reliability of the instrument, clarity of the questions, and respondents’ reaction to the questions and interviewers. Following the pre-test, some unclear questions were modified as required. When the head of the household (mother/father) was unavailable during the data collection period, repeated trials until three times was done. During the process of data collection, the supervisors and principal investigator regularly monitored and executed the overall activity to ensure the quality of data.

### Statistical analysis

The dependent variable in this study is traditional medicine utilization for children. The independent Variables include:

- Predisposing factors like family composition (age, sex, religion, economic level, education, family size, and residence status);
- Age of child;
- Sociocultural environment (accessibility, availability, cost, influence from peers/family, religious belief, cultural beliefs, the prevailing socio-cultural concept of illness);
- Perceived illness or sickness (perceived type and nature of the illness, perception of illness as serious, and experience of using for the previous child); and
- Health care experience of parents (parental TM use, dissatisfaction with modern medicine, the efficacy of TM and fear of side effects of modern medicine).

After data collection was finished; it was carefully double-checked, coded, entered, and cleaned using in Epi data version 3.1 and transferred to SPSS version 21.0 for further analysis. The exploratory logistic regression analysis was carried out to identify the cultural, demographic, and socioeconomic factors determining the use of TM. It was used to detect the association between the dependent variable and independent factors. Variables that demonstrated at least a trend towards univariate association with the outcome (defined as *P* < 0.2) were entered into the bivariate model. All tests were done at a two-sided alpha level of 5%.

Ethical clearance and approval to conduct this research were obtained from the Research and Ethical Review Committee of Department of Nursing and Midwifery, School of Allied Health Sciences, College of Health Sciences, Addis Ababa University. Moreover, the consents of District, Kebele, and respondents were obtained.

### Operational definitions

- **Herbal medicines:** are plants used for medical treatments, which are culturally acceptable.
- **Bone settler:** is a traditional practitioner who cures the balance of the skeleton, muscles and joint manipulation. He/she educates his/herself from tradition and takes up the practice of healing without having had any formal training.
- **Functional foods**: are foods that provide both physiological preventive and/or health-promoting effects to reduce the risk of chronic diseases and basic nutrition in the form of small solids or droplets. Functional foods include garlic, onion, ginger, orange juice, red pepper spice, pepper, to mention but a few.
- **Religious/prayer therapy:** are used in counseling to invite God’s healing presence to come and restore, forgive, erase, transform, and set free the inner life of the client to allow him or her to detach from sinful choices and painful trauma, and grow in all that Christ would have. *Tsefat* /*Kitab* and *Tsebel* can be cited as examples in the area.
- **Massage:** is the practice of applying gentle or strong pressure to the muscles and joints of the body to ease pain and tension using the hand, that is carried out by a locally reputable healer.
- **Parent:** includes father, mother or/and guardian who nurtures and raises a child.
- **Children:** are those who are < 18 years old.
- **Traditional healers/practitioners:** are healthcare providers who are not trained in modern medical science.
- **Traditional medicine utilization:** includes anything used in the promotion of health, prevention of illness and treatment of diseases and not currently considered to be part of modern medicine but accepted in that community. TM is neither prescribed by the healthcare professional nor used commonly as a diet in that particular culture.

## Results

### Socio-demographic characteristics

The survey has a response rate of 100%. The majority was Orthodox Christian religion followers and married. Greater than half of respondents could not read and write. The largest proportion are living in rural part of the District. The surveyed parents have a minimum of one child and maximum of 7 children. More than half of the participants have a monthly income of < 500 birrs/month i.e. about 23.3 USD. The socio-demographic characteristics of the parents are presented in Table 1**.**

**Table 1:** The table shows the socio-demographic characteristics of parents and children < 18 years old in Tole District, Oromia Regional State, and South-West Ethiopia

Variables		Frequency (***n*** = 267)	Percentage
**Sex of the Family**	Male	95	35.6
	Female	172	64.4
**Sex of a Child**	Male	151	56.6
	Female	116	43.4
**Age category of Parents**	20–29	76	28.5
	30–39	129	48.3
	40–49	45	16.8
	≥50	17	6.4
**Religion Affiliation**	Orthodox Christian	248	92.9
	Muslim	2	0.7
	Waqefata	7	2.6
	Protestant Christian	10	3.7
**Marital Status**	Married	251	94.0
	Single	3	1.1
	Widowed	6	2.2
	Divorced	7	2.6
**Educational status of the Family**	Not Read and Write	142	53.2
	Read and Write	14	5.2
	1st Cycle (Grade 1–4)	38	14.2
	2nd Cycle (Grade 5–8)	44	16.5
	High School (Grade 9–10)	16	6.0
	Preparatory (Grade 11–12)	6	2.2
	Tertiary Education	7	2.6
**No. of children < 18 years**	1–2	81	30.3
	3–4	136	50.9
	5–7	50	18.7
**Monthly income of household (Birr)**^**a**^	< 500	151	56.6
	> 500	116	43.4
**Residence**	Urban	85	31.8
	Rural	182	68.2

^a^Birr =0.045611USD or 1USD = 21.5 Birr during data collection

### Prevalence of traditional medicine utilization for children

The prevalence of TM practice for children was 212 (79.4%). Of these, 182 (85.9%) used at least one form of TM for their children in the last 12 months. The study depicted that about half (50%) of the parents obtained information on the benefit and efficacy of TM from their family members while some obtained from neighborhoods. It was surprising that only one person obtained information from a healthcare professional **(**Table 2**).**

**Table 2:** Prevalence and types of traditional medicine utilization for their children in the Tole District (*N* = 267)

Variables	Frequency	Percent
**Utilization of TM last 12 months (*n*** **= 212)**		
Yes	182	85.9
No	30	14.1
**When have you used TM for your child? (*n*** **= 212)**		
Within 1 month	31	14.6
Within 6 months	53	25.0
Before 6 months	128	60.4
**Reason to use TM for your previous child/children (*n*** **= 152)**		
When my child is sick	127	83.6
Use daily	4	2.6
Use weekly	2	1.3
When there is no improvement with modern medicine	10	6.6
As a preference to modern medicine	9	5.9
**Sources of information about the TM for your child (*n*** **= 212)**		
Self	9	4.2
Family	106	50.0
Relatives	20	9.5
Friends	6	2.9
Neighbors	34	16.0
Health professionals	1	0.5
Religious institutions	9	4.2
Traditional healers	27	12.7
**Type of TM ever used (*n*** **= 212)**		
Religious/prayer therapy	25	11.8
Herbal medicine	73	34.4
Bone settlers	16	7.5
Massage	55	25.9
Tooth extractor	13	6.1
Traditional Birth Attendant (TBA)	7	3.3
Functional foods	15	7.1
Other (*Tsefat* /*Kitab*)	8	3.9
**Source of TM (*n*** **= 152)**		
Cultivated	9	5.9
Wild	60	39.5
From healer	77	50.7
I don’t know	1	0.6
Prepared at home	5	3.3

### Type of traditional medicine utilization for their children

The herbal medicine, massage, and religious/prayer therapy for children were commonly used by the respondents. A quarter of the parents practiced TM for their children within the last 6 months while some have utilized TM for previous children. Most of them visited the nearby healers (Table 2).

**Fig. 1:**
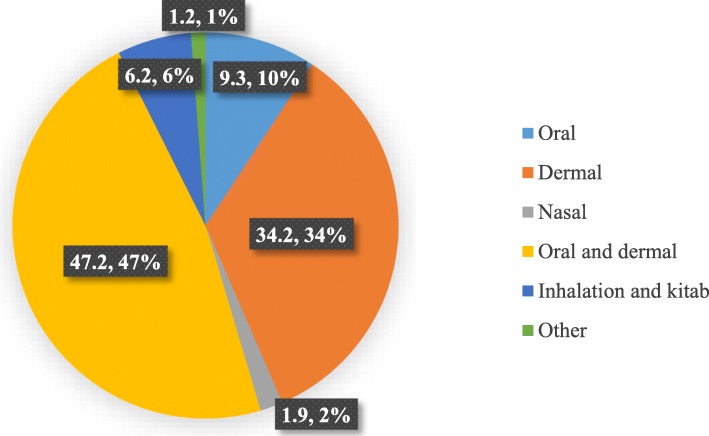
Route of TM they preferred for their children. Legend: The pie chart reveals the routes of TM administration that the parents prefer for their children. It could be orally, dermal, nasal, inhalations and *kitab*, and other routes

The study revealed that if the modern healthcare system was available and accessible in the localities, more than half 140 (52.5%) of the parents would have preferred to go to modern healthcare services while a few (3%) only chosen TM. Still, about 118 (44.2%) desired to use both modern and traditional medicine due to the costliness, experiences, and local beliefs as an alternative to unreachable and costly of the modern healthcare service delivery. In terms of mode of the administration, about 64(48%) of the respondents used both orally and dermally (Fig. 1).

### Reason for parental TM use for children

The parents used TM due to the socio-cultural, parental perception of illness/sickness, the parental experience with TM for themselves, and socio-demographic characteristics.

Socio-cultural reasons mainly include religious belief and ease of accessibility followed by cultural belief and costliness of the TM. Most parents consulted the traditional practitioners before administration of TM (Table 3).

**Table 3:** Socio-cultural, perception and experiences of parents on the utilization of traditional medicine for their children in the Tole District (*n* = 212)

Variables	Frequency	Valid Percent
**Reasons for using TM**		
Being easily accessible	118	55.7
Cost/cheap	114	53.8
Being referred by someone	95	44.8
Family influence	90	42.5
Cultural belief	114	53.8
Religious belief	121	57.1
**Experience and frequency of communicating with traditional healers**		
Never	36	17.0
Sometimes	71	33.5
As needed	89	42.0
Often	5	2.4
Very Often	11	5.2
**Duration of illness (*n*** **= 212)**		
Acute illness (1–30 days)	182	85.9
Chronic illness (> 30 days)	30	14.2
**Status of the child after treatment (*n*** **= 212)**		
Very Poor	2	0.9
Poor	5	2.4
Fair	52	24.5
Good	88	41.5
Very Good	65	30.7
**Family practice of TM for themselves**	**Frequency**	**Percent**
Yes	161	60.3
No	106	39.7
**Reason for applying TM compared to modern medicine (*n*** **= 161)**		
When selected correctly, it is effective	28	17.4
Satisfaction with Traditional Medicine	47	29.2
Dissatisfaction with modern medicine	19	11.8
The fear of using drugs and the side effects	8	5.0
Difficulty in accessing health care facilities/ costliness	15	9.3
Less efficacy of modern medicine	9	5.6
Knowledge of traditional medicine	35	21.7
**Level of satisfaction after TM utilization (*n*** **= 161)**		
Completely dissatisfied	7	4.3
Somewhat dissatisfied	14	8.7
Neutral	34	21.1
Somewhat satisfied	58	36.0
Completely satisfied	48	29.8

#### Perception of their illness/sickness

Most parents generally perceived that the health status of their children has improved after treatment with TM (Table 3).

### Experience of TM utilization for parents over the last 12 months

The family members also practiced traditional medicine for themselves over the last 12 months. Half of the mothers and about one-third of the fathers practiced TM because they were satisfied with and have knowledge about the particular TM. They rated TM efficacy as positive while the quality of the modern healthcare service was considered either as good or very good (Table 3).

**Fig. 2:**
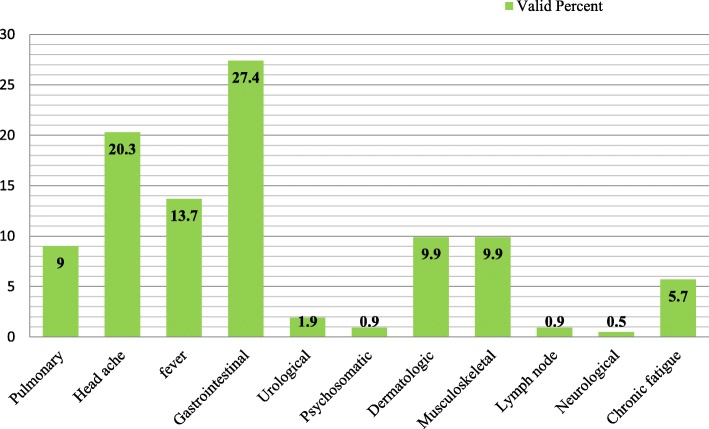
Perception of parents on child’s symptom and their respective treatment. Legend: The figure depicts the perception of parents on children’s symptoms and their respective treatment in the study area. The parents perceived pulmonary, headache, fever, gastrointestinal, urological, psychosomatic, dermatologic, musculoskeletal, lymph node, neurological, and chronic fatigues cases and used TM treatment for the same

The majority of parents 182(86%) used TM to treat illness and relieve symptoms; about 12% used to prevent illness in their children and only 2% used for the sake of promoting health conditions. Some parents consumed TM for the symptom of gastrointestinal (27.1%), headache (20.1%), and fever (13.7%) (Fig. 2).

### Factors associated with utilization of traditional medicine

The bivariate logistic regression analysis was employed to assess factors determining the utilization of TM and the association with parental TM use for children at *p*-value < 0.05 (95% C.I). Accordingly, the monthly household income, residence status, utilization of TM for the previous child, household income, ease of accessibility of TM, cultural influence, religious influence, parental TM use, and duration of illness were considered. These variables have shown associations with the utilization of TM in the last 12 months (Table 4).

**Table 4:** Factors associated with parental TM use for children in Tole District (*N* = 267)

Variables		Parental TM use for their children		COR (95%, CI)	AOR (95%, CI)	***P***-value
		Yes	No			
Monthly income of a household	< 500	154(57.7)	31(11.6)	1	1	
	> 501	**58(21.7)**	**24(9.0)**	**0.49(0.26, 0.90)**	**0.25(0.08, 0.78)**	0.021*
Residence	Urban	48(18.0)	37(13.8)	1		
	Rural	134(50.2)	48(18.0)	2.15(1.25, 3.70)		
Utilization of TM for the previous child	Yes	159(75.0)	21(9.9)	2.96(1.21, 7.25)		
	No	23(10.8)	9(4.2)	1		
Easily accessible	Yes	107(50.5)	11(5.2)	2.46(1.11, 5.48)		
	No	75(35.4)	19(9.0)	1	1	
Cultural influence	Yes	**105(49.5)**	**9(4.2)**	**3.18(1.38, 7.33)**	**3.01(1.16, 7.83)**	**0.024**
	No	**77(36.3)**	**21(9.9)**	**1**	**1**	
Religion influence	Yes	**110(51.8)**	**11(5.2)**	**2.64(1.17, 5.87)**	**3.17(1.26, 7.93)**	**0.014**
	No	**72(34.0)**	**19(9.0)**	**1**	**1**	
The parental practice of TM	Yes	128(60.4)	13(6.1)	2.20(1.30, 3.72)		
	No	54(25.5)	17(8.0)			
Duration of illness	Chronic(> 30 days)	**21(9.9)**	**9(4.2)**	1	**1**	
	Acute(< 30 days)	**161(75.9)**	**21(9.9)**	**3.29(1.33, 8.11)**	**3.11(1.07, 9.02)**	**0.037**

*COR* Cumulative Odd Ratio, *AOR* Adjusted Odd Ratio

The result depicted that parents who had higher monthly income (> 501) were less likely to use traditional medicine when compared to those who had low income (less than 500) [OR: 0.25(0.08, 0.78)]. The cultural influence was also significantly associated with parental TM practice for children. Parents who perceived TM due to cultural beliefs were 3.01 times more likely to use TM [OR = 3.01(1.16, 7.83)] for children than those did not belief. Moreover, parents who utilized TM for their children because of religious influence were 3.17 times higher compared to those who were not influenced by religious belief [OR = 3.17(1.26, 7.93)]. Similarly, parents who had an acute duration of illness (< 30 days) were 3.11 times to use TM for their children when compared to those who have a chronic duration of illness (≥30 days) [OR = 3.11(1.07, 9.02)].

## Discussions

Traditional medicine has increasingly gained popularity among parents for children in Ethiopian society [3]. In this study, the utilization of TM was found to be higher (about 85.9%) than the previous finding (around 80%) [16]. The higher prevalence in current study may be attributed to a strong relationship between the socio-cultural settings, as well as less accessibility to modern healthcare facilities in the area. Moreover, we found out that TM utilization was greater than the estimation of the National Center for Complementary and Integrative Health (NCCIH), which indicated the prevalence of using Traditional Medicine (TM) or Complementary and Alternative Medicine (CAM) in children between 9 and 73% globally [17]. This is because NCCIH studies were conducted in the healthcare setting while this study was conducted at the community level and relied on a huge perception assessment.

Among the studied parents, the most commonly used traditional medicines were herbal medicine, massage, religious therapy, bone settler, and therapy/prayer. The result concurred with a study from Northern part of Ethiopia, Motta Town, which depicted that the frequently used TMs for children were herbal medicine followed by religious/prayer practices, massage, and bone settler [3]. However, we did not observe any relationship between children’s type of illness/symptom and parental TM practice.

The most prevalent health problem was gastrointestinal, headache, fever, dermatological, musculoskeletal system, pulmonary disease, and chronic fatigue. Likewise, another study conducted in Ethiopia revealed that headache, gastrointestinal, dermatologic, chronic fatigue, and psychosomatic were frequently justified health problems to use TM [3]. The similarity suggested the prevalence of acute and communicable diseases among the Ethiopia society. Furthermore, a global context from the NCCIH substantiated that complementary health approaches were often used for back or neck pain, head or chest colds, anxiety or stress, other musculoskeletal problems, attention-deficit hyperactivity disorder, and insomnia for children [17].

The study also depicted that parental economic status was significantly associated with parental TM use for children. Parents with higher economic status were less likely to practice TM as they afford modern healthcare service even outside the District. Some studies came up with comparable findings. For example, in Jimma area of Ethiopia, most people went for cheap healthcare services such as TM [18]. In general, among the African community, the use of TM was related to low economic status [6]. Low level of income and preference for TM might be attributed to the incidence of poverty in the area, which made the affordability and accessibility of modern healthcare services very difficult.

The duration of illness was another factor that significantly associated with parental utilization of TM for their children. It was observed that TM was used three times more frequently for acute illness of any daily ailment than for chronic illness. The parents were driven to practice TM by the prevailing socio-cultural conditions of the family and the communities [14]. Likewise, the cultural belief of parents was significantly associated with and more often a motivating force of parental TM use for their children compared to those who did not believe. This is due to its acceptance in the community. Similar to our observation, a study from Jimma Zone of Ethiopia revealed that local people have been seeking for traditional herbal medicine even in preference to modern medications as a part of community’s belief. They believe that they would not get better remedy for some diseases in modern health services [18]. A neighboring country, Kenya, about 15% of the studied population trusted that herbal treatment is culturally well accepted in the community [7]. We have also seen that religious belief was a key factor that influenced the parental use of TM. Believers were 3.11 times more likely to practice TM compared to non-believers. This is also the case in Nigeria, where about 23% used TM for their children due to religious beliefs [19, 20]. Overall, the cultural acceptability and religious attitude of TM for some diseases in the community are strongly inculcated in everyday life.

Although parental use of TM for themselves was significantly associated with childhood TM practice in previous studies [14, 18, 19]; in this study, we haven’t observed any association between the utilization of TM for themselves and their children. Probably, this could be due to the government’s simultaneous focus on children and maternal health services. These services are being provided by health extension workers through an approach called community-based health education outreaches. As a result, they might hesitate to use TM for their children even though they used it for themselves.

The other surprising aspect of this study is the source of information for TM. The health professionals rarely provided information on TM to parents. Instead, they often encouraged the parents to bring their children to modern health centers. Yet, the use of TM is still prevalent. In a case where the availability of modern medical services is a hurdle either due to costliness or the parents are already using TM, the health professionals could advise the parents considering the effectiveness and the efficacy of the TM.

We would like to note that this study was based on the perception of the parents; it was not clinical-based experiment. As such, the safety and efficacy of TM was not established nor the subjective perception of the parents constituted standard health criteria to evaluate the same.

Given the scope of the study, it mainly focused on the utilization of TM for children. This can be taken as a limitation to be considered for further research. The other limitation of the study was its priority for mothers during interview on the assumption that mothers are closer to their children than fathers. In addition, the parental and children’s characteristics were not evaluated. In terms of the use of TM, there may be a risk of social desirability and some sort of subjectivity biases. Moreover, there was no possibility to identify whether TM practice affected the associated factors or whether there was association or effect between independent variables. However, in the process, maximum efforts were made to respect the culture of the community and stick to objectivity.

Finally, the study was cross-sectional and evaluated the effect of the variable of interest. In doing so, it contributed to previous studies and literature in Ethiopia, which are too tin and lacked the context and cultural specificity.

## Conclusion

There was high parental TM practice for children in this study. It indicated that the significant contribution of TM to public health. The study showed that for the majority of parents, TM was the key option to promote, prevent, and treat their children’s health/health problems. The most commonly used TM therapies were herbal medicine followed by massage and religious/prayer therapy. Monthly income of the household, cultural influence, religious belief, and duration of illness were associated with parental TM use for their children. Therefore, three issues need to be taken into account. First, since there was a high prevalence of TM use in the community, the health professional should closely counsel and device a controlling mechanism of traditional healers and TM user-friendliness. To this end, extension workers must educate, support and counsel community in general and the women in particular. Second, in order to expand TM, safety and efficacy should be carefully studied through further national-wide research, preferably using mixed approach, taking into account the children’s characteristics. Third, the modern medical services should be expanded both in terms of affordability and accessibility hand-in-hand with promoting TM utilization.

## Supplementary information

**Additional file 1.**

## Data Availability

The datasets used and/or analyzed during the current study are available from the corresponding author via email.

## Abbreviations

CAM: Complementary and Alternative Medicine
CI: Confidence Interval
HEWs: Health Extension Workers
NCCIH: National Center for Complementary and Integrative Health
COR: Cumulative Odd Ratio
AOR: Adjusted Odd Ratio
SPSS: Statistical Package for Social Science
TBA: Traditional Birth Attendant
TM: Traditional Medicine
WHO: World Health Organization
