# Supplementary material for: Determinants of traditional medicine utilization for children: a parental level study in Tole District, Oromia, Ethiopia
Source: BMC Complement Med Ther. 2020 Apr 23;20:125. doi: 10.1186/s12906-020-02928-1 (PMC7178580; doi:10.1186/s12906-020-02928-1)
Supplement: Supplementary file 1 — Additional file 1. [file 12906_2020_2928_MOESM1_ESM.docx]

# APPENDIX 1: CONSENT FORM

Addis Ababa University

College of Health Sciences

School of Allied Health Sciences

Department of Nursing and Midwifery

I here with declare that:

- The objectives of this study are explained to me and are clear.
- The contents of the consent are verified to me to participate in the study.

I understand that participation in this study is completely voluntary and that I may withdraw at any time without supplying reasons. I agree to participate in this study to be interviewed, provided my privacy is guaranteed. When signing this consent form to participate in the study, I promise to answer honestly to all reasonable questions and not provide any false information or in any other way purposely mislead the researcher.

Signature of the participant_______________________ date ____________________ Signature of the investigator_______________________ date ____________________

# APPENDIX 2: QUESTIONNAIRE, ENGLISH VERSION

**Section I: Socio-Demographic Characteristics of the Parents**

**Instruction:** Please circle the number in front of the option you choose.

| **N_O_.** | **Variable** | **Coding categories** |
| --- | --- | --- |
| 101 | Sex | 1. Male 2. Female |
| 102 | Age(in years) | 1. __________ |
| 103 | What is your religious affiliation? | 1. Orthodox 2.Muslim 2. Wakefata 3. Protestant 3. Others |
| 104 | Marital status | 1. Married 3. Single 2. Divorced 4.Widowed |
| 105 | Educational status | 1. No read and write 2. Read and write 3. 1^st^. cycle (1-4) 4. 2^nd^ cycle (5-8) 5. High school (9-10) 6. Preparatory (11-12) 7. Tertiary education |
| 106 | How far from the nearest health care center? | __________ |
| 107 | How many children do you have (up to 18 years)? | __________ |
| 108 | Monthly income level(ETB) | ____________ |
| 109 | Residence | 1. Urban 2. Rural |
| 110 | Age of the participating child in years | ____________ |
| 111 | Sex of the child | 1. Male 2.Female |
| 112 | Educational status of the child | 1. No read and write 2. Read and write 3. 1^st^. cycle (1-4) 4. 2^nd^ cycle (5-8) 5. High school (9-10) 6. Preparatory (11-12) |

**Section II: Traditional Medicine practice**

**Instruction:** Please circle the number in front of the option you choose.

| **N_O_.** | **Variable** | **Coding category** |
| --- | --- | --- |
| 113 | Have you ever used Traditional Medicine for your child? | 1. Yes 2. No |
| 114 | If ‘yes’ to question number 113, have you used Traditional Medicine for your child with in the last 12 months? | 1. Yes 2. No   99. Not Applicable |
| 115 | Have you ever used any of the following complementary or traditional therapies? | 1. Religious/prayer therapy 2. Herbal medicine 3. Bone settlers 4. Massage 5. Tooth extractor 6. TBA 7. Functional foods 8. Any others (specify)… |
| 116 | When have you used Traditional Medicine for your child? | 1. Within 1 month 2. Within 6 months 3. Before six months |

**Section-III Type of tradition medicine used for their children**

**Instruction:** Please circle the number in front of the option you choose.

| **No** | **Variable** | **Coding category** |
| --- | --- | --- |
| 117 | Which health care service do you prefer? | 1. Modern 3. Traditional 2. Both 4. No opinion |
| 118 | Are you practicing traditional medicine for your previous child? | 1. Yes 2. No |
| 119 | Which one used for your previous child? | 1. Religious/prayer therapy 2. Herbal medicine 3. Bone settlers 4. Massage 5. Tooth extractor 6. Traditional Birth Attendants 7. Functional foods 8. Any others (specify)…………… |
| 120 | If your response for question No. 119 is ‘Yes’, when do you use the above traditional medicine for your child? | 1. My child sick 2. Use daily   3.Use weekly 4.Monthly   1. No improvement with modern medicine 2. Always used as food, prophylaxis 3. Prefer than traditional medicine |
| 121 | From where you get traditional medicine? | 1. Cultivated 3. Wild 2. From healer 4. I don’t know   5.Other________ |
| 122 | Which route do you prefer? | 1. Oral 3.Dermal 2. Nasal 4. Oral and dermal   5.Other, specify ----------------------------- |

**Section IV: socio-cultural environment for preference and utilization of traditional medicine.**

**Instruction:** Please circle the number in front of the option you choose.

| **N_O_.** | **Variable** | **Coding category** |
| --- | --- | --- |
| 123 | What is your reasons for using Traditional Medicine | 1. Being easily accessible 2. Cost/cheap in price 3. Being referred by someone 4. Family influence 5. Cultural belief 6. Religious belief 7. Any others? (specify)------- |
| 124 | What are your sources of information about the traditional medicine for your child is using? | 1. Self 6. Family 2. Relative 7. Friends 3. Neighbors 8.Health professionals 4. Religious institutions 9.Traditional healers 5. Media 10. Any other? Specify--------- |
| 125 | How much were you personally able to communicate about your child’s health with the person(s) who provided the therapy for you? | 1. Never 4. Some Times 2. As Needed 5. Often 3. Very Often |

**Section V: perceived illness/sickness**

**Instruction:** Please circle the number in front of the option you choose.

| **N_O_.** | **Variable** | **Code category** |
| --- | --- | --- |
| 126 | For what purpose you used Traditional Medicine for your child? | 1. To promote health 2. To prevent illness 3. To treat illness/symptom relief 4. Others (specify)……… |
| 127 | In your perception, the overall health status of your child before treatment is | 1.Very poor 2.Poor 3.Fair 4.Good 5.Very good |
| 128 | To what extent do you feel that pain or discomfort prevents your child from doing everyday things you need to do? | 1.Not at all 2.Little  3.Moderate 4.High 4.Very high |
| 129 | For which child’s symptom you used traditional medicine? | 1. Pulmonary 7. Gastrointestinal 2. Urological 8. Psychosomatic 3. Dermatologic 9. Musculoskeletal 4. Lymph node 10.Neurological 5. Chronic fatigue 11. Head ache   12.Any others? (Specify)… |
| 130 | What is the duration of illness? | __________________ |
| 131 | What does the overall health status of your child after treatment looks like? | 1. Very poor 2.Poor   3.Fair 4.Good 5.Very good |

**Section VI: Healthcare Experience**

**Instruction:** Please circle the number in front of the option you choose.

| **N_O_.** | **Variable** | **Coding category** |
| --- | --- | --- |
| 132 | Have you ever used Traditional Medicine for yourself within the last 12 months? | 1. Yes 2. No |
| 133 | If your response is ‘yes’ to question No.129, who had used Traditional Medicine? | 1. Mother only 2.Father only   3.Both 4.Grand parents  5.Others, specify -----------------------------  99.Not applicable |
| 134 | What is (are) reason(s) for applying Traditional Medicine than modern medicine? | 1. When selected correctly it is effective 2. Satisfaction with Traditional Medicine 3. Dissatisfaction with modern medicine 4. The fear of using drugs and the side effects 5. Difficulty in accessing health care facilities/ high cost 6. Less efficacy of modern medicine 7. Knowledge of traditional medicine 8. Any other? Specify…… |
| 135 | Would you please rate the level of Traditional Medicine efficacy? | 1.Very poor 2.Poor 3.Fair  3. Good 4.Very good |
| 136 | Would you please rate your level of satisfaction after traditional medicine use? | 1. Completely dissatisfied 2. Somewhat dissatisfied 3. Neither satisfied nor dissatisfied 4. Somewhat satisfied 5. Completely satisfied |
| 137 | Overall, how do you rank the quality of your experience with modern healthcare systems? | 1. Very bad 2. Bad 3.Moderate   4. Good 5.Very good |
| 138 | Could you please tell me anything relevant to healthcare problems in your area? | ____________ |

**Thank you for your time!!!**
